# Association Between Thiazolidinedione Therapy and Tuberculosis-Related Events in Type 2 Diabetes: A Population-Based Cohort Study

**DOI:** 10.3390/microorganisms14091979

**Published:** 2026-09-08

**Authors:** Fu-Shun Yen, James Cheng-Chung Wei, Shao-Fan Yen, Chen-Yu Sung, Teng-Shun Yu, Fuu-Jen Tsai, Chih-Cheng Hsu, Chii-Min Hwu

**Affiliations:** 1 Dr. Yen’s Clinic, No. 15, Shanying Road, Gueishan District, Taoyuan 33354, Taiwan; yenfushun@gmail.com; 2 Department of Allergy, Immunology & Rheumatology, Chung Shan Medical University Hospital, No. 110, Sec. 1, Jianguo N. Rd., South District, Taichung 40201, Taiwan; 3 Institute of Medicine, Chung Shan Medical University, Taichung 40201, Taiwan; 4 Graduate Institute of Integrated Medicine, China Medical University, Taichung 40402, Taiwan; 5 Brunel Medical School, Brunel University of London, Kingston Lane, Uxbridge, Middlesex UB8 3PH, UK; 6 Management Office for Health Data, China Medical University Hospital, Taichung 40201, Taiwan; 7 College of Medicine, China Medical University, Taichung 40402, Taiwan; 8 School of Chinese Medicine, College of Chinese Medicine, China Medical University, Taichung 40402, Taiwan; 9 Department of Medical Research, China Medical University Hospital, Taichung 40402, Taiwan; 10 Division of Medical Genetics, China Medical University Children’s Hospital, Taichung 40447, Taiwan; 11 Department of Biotechnology and Bioinformatics, Asia University, Taichung 41354, Taiwan; 12 Institute of Population Health Sciences, National Health Research Institutes, 35 Keyan Road, Miaoli County 35053, Taiwan; 13 Department of Health Services Administration, China Medical University, Taichung 40402, Taiwan; 14 Department of Family Medicine, Min-Sheng General Hospital, 168 ChingKuo Road, Taoyuan 33044, Taiwan; 15 National Center for Geriatrics and Welfare Research, National Health Research Institutes, No. 8, Xuefu W. Rd., Yunlin County 632007, Taiwan; 16 Faculty of Medicine, National Yang Ming Chiao Tung University School of Medicine, No. 155, Sec.2, Linong Street, Taipei 11221, Taiwan; 17 Section of Endocrinology and Metabolism, Department of Medicine, Taipei Veterans General Hospital, No. 201, Sec. 2, Shipai Road, Beitou District, Taipei 11217, Taiwan

**Keywords:** thiazolidinedione, rosiglitazone, pioglitazone, tuberculosis, pulmonary TB, TB-related hospitalisation

## Abstract

Tuberculosis (TB) remains a major global health burden, and diabetes mellitus increases the risk of active TB. Thiazolidinediones (TZDs) have immunomodulatory effects, but their association with TB remains unclear. This study investigated the associations between TZD use and TB-related events in patients with type 2 diabetes (T2D), including TB requiring anti-TB treatment, pulmonary TB, and TB-related hospitalization. Using Taiwan’s National Health Insurance Research Database, adults with newly diagnosed T2D between 2000 and 2017 were identified. Propensity score matching and Cox proportional hazards models were used to estimate adjusted hazard ratios (aHRs) and 95% confidence intervals (CIs) for TB requiring anti-TB treatment, pulmonary TB, and TB-related hospitalization. After 1:1 matching, 44,095 TZD user–nonuser pairs and 9225 rosiglitazone user–nonuser pairs were identified. Overall, TZD use was not significantly associated with TB-related outcomes. However, rosiglitazone use was associated with lower risks of TB requiring anti-TB treatment (aHR, 0.56; 95% CI, 0.36–0.86), pulmonary TB (aHR, 0.79; 95% CI, 0.68–0.92), and TB-related hospitalization (aHR, 0.59; 95% CI, 0.43–0.81). Rosiglitazone use was associated with lower risks of TB-related outcomes in the primary Cox analyses; however, these associations were attenuated in time-dependent analyses and were no longer statistically significant after accounting for death as a competing event. These observational findings should therefore be considered hypothesis-generating rather than evidence of a causal or protective effect and require confirmation using more robust causal-inference approaches.

## 1. Introduction

Tuberculosis (TB) is an airborne infectious disease caused by the *Mycobacterium tuberculosis* (*M. tuberculosis*) complex. Although it primarily affects the lungs, it can involve nearly any organ. After exposure, host immunity may confine bacilli within granulomas, resulting in latent infection; failure of containment may lead to active pulmonary TB with clinical manifestations and transmissibility [1]. TB remains one of the most prevalent infectious diseases worldwide. In 2024, an estimated 10.7 million individuals developed TB, corresponding to an incidence of 131 per 100,000 population [2]. TB was also the leading cause of death from a single infectious agent, accounting for approximately 1.23 million deaths and surpassing both COVID-19 and HIV/AIDS [2]. In Taiwan, 6141 new TB cases were reported in 2024 (26.2 per 100,000 population), with 427 TB-related deaths (1.8 per 100,000 population) [3]. Beyond its clinical impact, TB also imposes substantial socioeconomic and psychosocial burdens, including financial hardship, work disruption, stigma, and social isolation [4]. Despite ongoing control efforts, the global decline in TB incidence remains insufficient to meet WHO End TB Strategy targets [2,5].

Diabetes mellitus is an important obstacle to TB control. Individuals with diabetes have an approximately threefold increased risk of developing active TB and experience poorer treatment outcomes, including higher risks of mortality, treatment failure, and relapse [6,7,8]. In 2013, nearly one million TB cases worldwide were attributable to diabetes [9]. As diabetes prevalence continues to rise globally, its impact may substantially hinder future reductions in TB incidence [9,10,11]. Therefore, effective TB prevention strategies for individuals with diabetes are urgently needed.

Chronic hyperglycemia impairs innate immune function through the accumulation of advanced glycation end products and reactive oxygen species, reducing the ability of neutrophils and macrophages to eliminate pathogens such as *M. tuberculosis* [12]. Thiazolidinediones (TZDs), including rosiglitazone and pioglitazone, are peroxisome proliferator-activated receptor-γ (PPARγ) agonists that improve insulin sensitivity and exert anti-inflammatory and immunomodulatory effects [13,14]. Despite belonging to the same drug class, the two agents are pharmacologically distinct: rosiglitazone is a more selective and potent PPARγ agonist, whereas pioglitazone also exhibits partial PPARα activity [15]. These differences in receptor activity may result in distinct metabolic and immunomodulatory effects. Experimental studies suggest that PPARγ activation can regulate macrophage function, suppress proinflammatory cytokines, and modulate immune-cell activity [14]. However, this immunomodulation presents a potential biological paradox: although limiting excessive inflammation may reduce tissue injury, excessive suppression of inflammatory responses could theoretically impair protective host immunity against M. tuberculosis. Thus, both class-wide and agent-specific effects of TZDs on TB susceptibility are biologically plausible but remain uncertain. Clinical evidence regarding the association between TZD use and TB risk remains scarce and inconsistent. One previous study reported a significantly lower risk of incident pulmonary TB among TZD users [16], whereas another found only a nonsignificant reduction in TB risk among patients with higher TZD exposure [17]. Thus, the association between TZD therapy and TB risk remains uncertain. An important unresolved question is whether any potential association between TZD therapy and TB risk is mediated primarily by improved glycemic control and reversal of hyperglycemia-associated immune dysfunction, or by more direct PPARγ-mediated immunomodulatory effects. Therefore, this nationwide cohort study evaluated the associations between overall TZD use and TB-related events and examined rosiglitazone and pioglitazone separately, while considering whether the observed associations might be compatible with metabolic or drug-specific immunomodulatory mechanisms.

## 2. Materials and Methods

### 2.1. Study Design and Data Source

A nationwide retrospective cohort study was conducted using Taiwan’s National Health Insurance Research Database (NHIRD), which covers more than 99% of Taiwan’s population and contains longitudinal claims data, including demographics, diagnoses, procedures, and prescriptions. Diagnoses were coded using ICD-9-CM and ICD-10-CM, and all personal identifiers were encrypted before release [18]. The study was approved by the Research Ethics Committee of China Medical University and Hospital (CMUH112-REC1-117[CR-3]), which waived the requirement for informed consent because de-identified data were used. This study followed the STROBE guidelines for cohort studies [19].

### 2.2. Participants

Adults aged 20–80 years with newly diagnosed type 2 diabetes (T2D) between 1 January 2000 and 31 December 2017 were identified. T2D was defined by at least two outpatient claims or one inpatient claim with T2D diagnostic codes and receipt of at least one antidiabetic medication. The validity of diabetes diagnoses in Taiwan’s National Health Insurance claims database has been previously evaluated [20].

**Figure 1:**
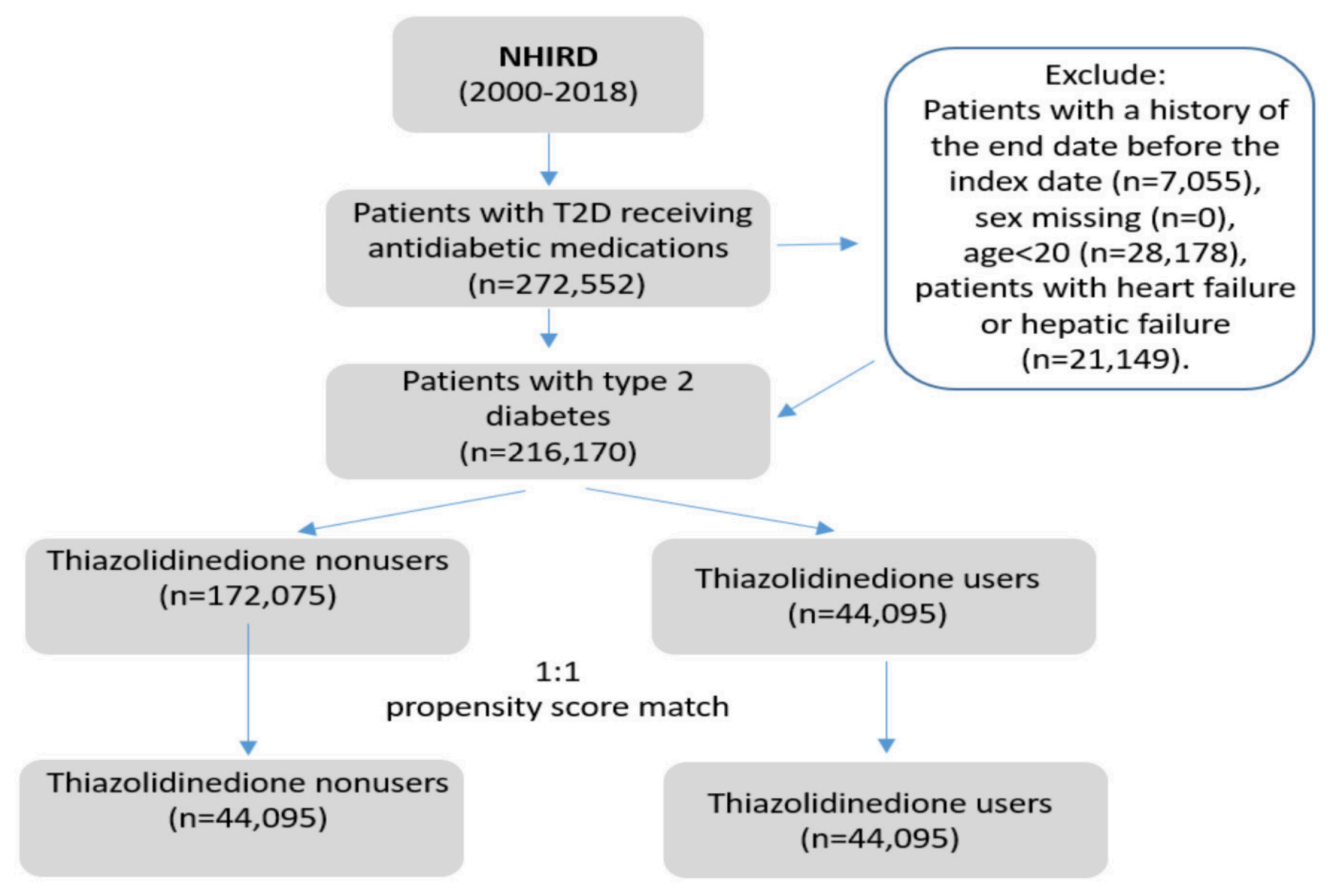
Flow chart of the identified process in this study.

Participants were excluded if they had TB before the index date (n = 7055), heart failure, hepatic failure (n = 21,149), missing demographic information (n = 0), or were younger than 20 years (n = 28,178). Cohort selection is illustrated in Figure 1.

### 2.3. Treatment Assignment and Comparison Groups

Exposure status for the primary analysis was assigned at cohort entry using an intention-to-treat (ITT)-like approach. For TZD users, the index date was the date of the first TZD prescription. For matched nonusers, the index date was assigned by applying the same elapsed time from T2D diagnosis to the index date as that observed in the corresponding TZD user. This index-date assignment also aligned the duration from T2D diagnosis to cohort entry between matched users and nonusers, thereby reducing potential confounding by diabetes duration. In the primary analysis, participants retained their initial exposure classification throughout follow-up regardless of subsequent treatment discontinuation, switching between rosiglitazone and pioglitazone, or initiation of TZD therapy among baseline nonusers; therefore, no censoring was applied for these treatment changes. Follow-up continued until the occurrence of a study outcome, death, withdrawal from the insurance system, or the end of the study period. This approach preserved baseline treatment assignment but may have introduced exposure misclassification when treatment changed during follow-up.

### 2.4. Covariates

Potential confounders were selected a priori based on clinical relevance and prior literature. These included demographic characteristics (age and sex), index year, lifestyle-related conditions were assessed using diagnosis-based proxies available within the claims database, including obesity and smoking-related diagnoses, and comorbidities identified within 1 year before the index date, including alcohol-related disorders, hypertension, dyslipidemia, coronary artery disease, peripheral arterial disease, stroke, chronic obstructive pulmonary disease (COPD), chronic kidney disease, liver cirrhosis, rheumatologic diseases, malignancy, psychotic disorders, depression, dementia, and organ transplantation, as well as baseline clinical events such as prior pneumonia. A history of pneumonia was included as a marker of underlying respiratory health and a potential confounder for subsequent tuberculosis risk. Medication use was assessed during the 1-year period preceding the index date and included oral antidiabetic drugs, insulin, statins, aspirin, systemic corticosteroids, and immunosuppressants. Baseline medication use was defined as at least one prescription for the corresponding medication during this 1-year look-back period, rather than medication use specifically on the index date. Thus, the relatively high proportions of insulin and systemic corticosteroid use reflect any recorded exposure during the entire baseline year and should not be interpreted as continuous or concurrent use at cohort entry. Disease burden indices were evaluated using the Charlson Comorbidity Index (CCI) and the Diabetes Complication Severity Index (DCSI) [21,22] (Table 1 and Table S2).

### 2.5. Outcomes

The study outcomes were (1) pulmonary TB, (2) TB plus receipt of anti-TB treatment, and (3) TB-related hospitalization, representing different levels of diagnostic certainty and disease severity. Pulmonary TB was identified using diagnostic codes, whereas TB plus receipt of anti-TB treatment was designated as the primary high-specificity endpoint and required both a TB diagnosis and a prescription for anti-TB medication. TB-related hospitalization was used as an indicator of more severe disease. This combined diagnosis-and-treatment definition has been validated in the NHIRD, demonstrating high sensitivity (96.3%) and excellent agreement (κ = 1.00) [23]. However, the original claims-based algorithm did not require a specific multidrug regimen or minimum treatment duration; therefore, some misclassification of latent TB preventive therapy, particularly isoniazid- or rifampicin-based treatment, cannot be completely excluded. Participants were followed until the first occurrence of a study outcome, death, or 31 December 2018.

### 2.6. Statistical Analysis

Propensity scores were estimated using multivariable logistic regression models incorporating 44 baseline characteristics, including age, sex, index year, comorbidities, diabetes severity, and concomitant medications. Index year was included to reduce confounding arising from temporal changes in rosiglitazone prescribing, reimbursement policies, and cardiovascular safety restrictions. Patients were matched using nearest-neighbor matching without replacement. Covariate balance before and after matching was assessed using standardized mean differences (SMDs), with absolute SMDs < 0.1 indicating adequate balance. Covariate balance was also visually assessed using Love plots (Figure S1). Approximate 1-year numbers needed to treat (NNTs) were calculated as the reciprocal of the absolute incidence-rate difference between rosiglitazone users and nonusers (1000/absolute rate difference per 1000 person-years). Associations between TZD exposure and TB outcomes were evaluated using Cox proportional hazards models and reported as hazard ratios (HRs) with 95% confidence intervals (CIs). The proportional hazards assumption was formally evaluated using Schoenfeld residuals for all Cox proportional hazards models. No substantial violations of the proportional hazards assumption were detected. Kaplan–Meier methods and log-rank tests were used to compare cumulative incidence between groups. To examine duration–response relationships, cumulative rosiglitazone exposure was categorized as 1–84, 85–420, and >420 days and compared with nonuse. To further characterize the exposure–response relationship without imposing categorical cutoffs, restricted cubic spline analyses were performed using cumulative rosiglitazone exposure as a continuous variable to evaluate potential nonlinear or threshold associations with TB-related outcomes.

Several measures were implemented to reduce potential bias, including alignment of index dates to minimize immortal time bias, propensity score matching to address confounding by indication, exclusion of individuals with prior TB or TB events within six months of the index date to reduce reverse causation, and use of validated outcome definitions to limit misclassification. Selection bias was further minimized by including a nationwide population. Prespecified subgroup analyses were conducted according to age, sex, COPD, CCI, DCSI, oral antidiabetic medication use, insulin use, and corticosteroid use. Time-dependent analyses were also performed. This analysis was presented separately from the primary ITT-like baseline-fixed analysis. Because participants were not censored specifically at treatment discontinuation or switching, this time-dependent analysis should not be interpreted as a strict as-treated analysis. Because death precludes the subsequent occurrence of TB and may therefore influence estimates of TB risk, competing-risk analyses were conducted using Fine–Gray subdistribution hazard models, with death treated as a competing event. Subdistribution hazard ratios (sHRs) and 95% CIs were estimated for each TB-related outcome.

All tests were two-sided, and a *p*-value < 0.05 was considered statistically significant. Because secondary, duration, subgroup, and sensitivity analyses were exploratory, no formal adjustment for multiple comparisons was applied; therefore, findings from these analyses should be interpreted cautiously in view of the potential for type I error. Analyses were performed using SAS 9.4 and Stata SE 11.0.

## 3. Results

### 3.1. Baseline Characteristics of the Study Participants

Between 1 January 2000 and 31 December 2017, 272,552 individuals with newly diagnosed T2D receiving antidiabetic therapy were identified. After exclusions, 44,095 TZD users and 172,075 nonusers remained (Figure 1). Following 1:1 propensity score matching, 44,095 matched pairs of TZD users and nonusers were generated (Table S2). In addition, 9225 matched pairs of rosiglitazone users and nonusers were established. Baseline characteristics were well balanced, with all standardized mean differences < 0.1 (Table 1 and Table S2). Love plots illustrating covariate balance before and after matching are presented in Figure S1. In the matched rosiglitazone cohort, 51.35% were men and the mean (SD) age was 61.13 (11.55) years. Mean (SD) follow-up durations were 9.64 (5.13) years for rosiglitazone users and 8.40 (5.49) years for nonusers.

### 3.2. TZD Use and Risk of Tuberculosis

In the matched TZD cohort, overall TZD use was not significantly associated with TB plus receipt of anti-TB treatment, pulmonary TB, or TB-related hospitalization compared with nonuse (Table S3). However, pioglitazone use was associated with an increased risk of TB plus receipt of anti-TB treatment (aHR 1.43, 95% CI 1.18–1.74), whereas rosiglitazone use was associated with lower risks of TB plus receipt of anti-TB treatment (aHR 0.57, 95% CI 0.44–0.74) and TB-related hospitalization (aHR 0.66, 95% CI 0.52–0.84). The association with pulmonary TB was not statistically significant.

### 3.3. Primary Analysis: Rosiglitazone Use and Tuberculosis Outcomes

In the dedicated propensity score-matched rosiglitazone cohort, rosiglitazone use was associated with lower risks of TB plus receipt of anti-TB treatment (aHR 0.56, 95% CI 0.36–0.86), pulmonary TB (aHR 0.79, 95% CI 0.68–0.92), and TB-related hospitalization (aHR 0.59, 95% CI 0.43–0.81) (Table 2). Absolute incidence-rate reductions were 0.29, 1.22, and 0.46 cases per 1000 person–years, corresponding to approximate 1-year NNTs of 3448, 820, and 2174 for TB plus receipt of anti-TB treatment, pulmonary TB, and TB-related hospitalization, respectively. Despite the relative risk reductions, the absolute differences were modest and the corresponding NNTs were large, reflecting the low incidence of TB-related outcomes. The higher incidence of pulmonary TB likely reflects the broader sensitivity of diagnosis-code-based ascertainment, which may capture provisional or suspected pulmonary TB cases that were subsequently not confirmed or did not receive anti-TB treatment. In contrast, requiring both a TB diagnosis and anti-TB treatment provides a more specific definition of clinically treated active TB. Because microbiological and radiographic data were unavailable, it could not be determined whether this discrepancy reflected unconfirmed suspected cases, differences in diagnostic coding practices, or true overdiagnosis.

**Figure 2:**
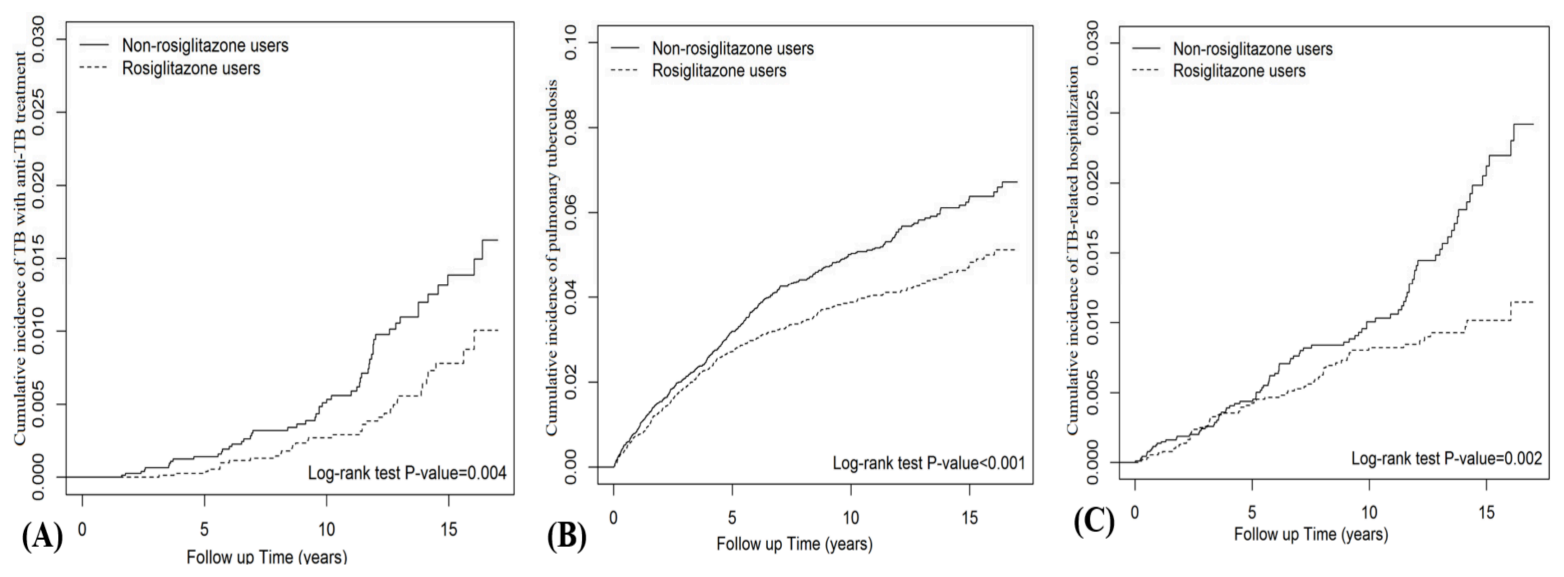
Cumulative incidences of (**A**) TB plus receipt of anti-TB treatment, (**B**) pulmonary tuberculosis, (**C**) TB-related hospitalization for rosiglitazone users vs. nonusers. Note: TB, tuberculosis.

Kaplan–Meier analyses further demonstrated significantly lower cumulative incidence among rosiglitazone users for all three outcomes (log-rank *p* = 0.004, *p* < 0.001, and *p* = 0.002, respectively; Figure 2), supporting the temporal consistency of these findings.

### 3.4. Cumulative Duration of Rosiglitazone Exposure

Cumulative duration analyses showed heterogeneous patterns across outcomes (Table 3). A more consistent decrease in risk across increasing exposure categories was observed for pulmonary TB, whereas no clear monotonic pattern was evident for TB plus receipt of anti-TB treatment or TB-related hospitalization. Although linear-trend tests yielded *p* values of 0.0239, <0.001, and 0.0116, respectively, the categorical estimates and restricted cubic spline analyses (Figure S2) did not support a uniformly monotonic exposure–response relationship across all outcomes. These findings should therefore be considered exploratory.

### 3.5. Sensitivity and Competing-Risk Analyses

After excluding patients with TB-related events within 6 months of the index date, rosiglitazone users remained at lower risk of TB plus receipt of anti-TB treatment (aHR 0.66, 95% CI 0.49–0.89), pulmonary TB (aHR 0.60, 95% CI 0.46–0.78), and TB-related hospitalization (aHR 0.59, 95% CI 0.44–0.80) (Table S4). In the complementary time-dependent exposure analysis, the associations for TB plus receipt of anti-TB treatment and pulmonary TB were attenuated and no longer statistically significant, whereas the association with a lower risk of TB-related hospitalization remained significant (aHR, 0.49; 95% CI, 0.24–0.99) (Table S5). Importantly, the associations were substantially attenuated when death was accounted for as a competing event using Fine–Gray subdistribution hazard models (Table 4). Rosiglitazone use was not significantly associated with TB plus receipt of anti-TB treatment (sHR, 1.00; 95% CI, 0.76–1.32), pulmonary TB (sHR, 0.90; 95% CI, 0.71–1.15), or TB-related hospitalization (sHR, 0.86; 95% CI, 0.65–1.13). Thus, none of the associations observed in the primary Cox analyses remained statistically significant after accounting for the competing risk of death, indicating that mortality materially influenced the estimated associations.

Subgroup analyses showed that the associations between rosiglitazone use and TB-related outcomes varied across patient characteristics (Tables S6–S8 and Figures S3–S5). For TB plus receipt of anti-TB treatment, significantly lower risks were observed among patients aged < 65 years, those without COPD, those with DCSI ≥ 2, and those without corticosteroid use. For pulmonary TB, significantly lower risks were observed among patients aged ≥ 65 years, patients with or without COPD, both DCSI strata, and patients with or without corticosteroid use. For TB-related hospitalization, significantly lower risks were observed across both age groups and COPD strata, among patients with DCSI ≥ 2, and irrespective of corticosteroid use. These subgroup findings should be interpreted as exploratory.

## 4. Discussion

In this nationwide cohort study, overall TZD use was not significantly associated with TB plus receipt of anti-TB treatment, pulmonary TB, or TB-related hospitalization compared with nonuse. In contrast, rosiglitazone, but not pioglitazone, was associated with significantly lower risks of TB plus receipt of anti-TB treatment and TB-related hospitalization after multivariable adjustment. A dedicated propensity score–matched analysis further showed lower risks of all three TB-related outcomes among rosiglitazone users. However, these associations were not consistently reproduced in sensitivity analyses. In the time-dependent analysis, the associations with TB plus receipt of anti-TB treatment and pulmonary TB were attenuated and no longer statistically significant, and all three associations became nonsignificant when death was treated as a competing event. Notably, the association with TB plus receipt of anti-TB treatment shifted from a lower risk in the conventional Cox analysis (aHR, 0.56) to the null in the Fine–Gray model (sHR, 1.00). Importantly, the attenuation of the associations in the time-dependent analyses and their disappearance after accounting for death as a competing event indicate that the primary Cox estimates may have been materially influenced by differential survival, survivor/selection bias, and treatment-selection effects. Therefore, the primary findings should not be interpreted as evidence that rosiglitazone protects against TB, but rather as hypothesis-generating associations requiring confirmation using study designs that more rigorously address time-varying exposure, competing mortality, and residual confounding.

Several glucose-lowering agents have previously been associated with reduced TB risk in patients with T2D, including metformin [24,25], α-glucosidase inhibitors [17], DPP-4 inhibitors [18], and SGLT2 inhibitors [26]. Evidence regarding TZDs, however, has been inconsistent. One study reported a significantly lower risk of incident pulmonary TB among TZD users [16], whereas another found only a nonsignificant reduction in TB risk among patients with higher TZD exposure [17]. One possible explanation is that previous studies generally evaluated TZDs as a homogeneous drug class, combining rosiglitazone and pioglitazone despite their pharmacological differences. Such class-level analyses may dilute or obscure agent-specific associations. Consistent with this possibility, overall TZD use showed no significant association with TB-related outcomes, whereas rosiglitazone and pioglitazone exhibited different patterns. The cumulative duration analyses also provided only limited evidence of an exposure–response relationship. Although pulmonary TB showed a more consistent decline in risk with increasing exposure, the other outcomes did not demonstrate clear monotonic patterns. Because cumulative exposure was analyzed as a time-fixed variable, these findings are susceptible to immortal-time, survivor, and healthy-adherer biases and should therefore be considered exploratory rather than evidence of a causal dose–response relationship.

Rosiglitazone has a complex cardiovascular safety history. Concerns regarding an increased risk of myocardial infarction led to prescribing restrictions after a 2007 meta-analysis, although subsequent evidence from the RECORD trial and its independent re-adjudication did not confirm a significant increase in myocardial infarction or cardiovascular mortality, leading the U.S. FDA to remove these restrictions in 2013 [27]. Nevertheless, TZDs, including rosiglitazone, can cause fluid retention and increase the risk of heart failure, which remains an important clinical consideration. Any potential association with lower TB risk must therefore be weighed against these established safety concerns. Moreover, given the modest absolute risk differences and the attenuation of the associations in time-dependent and competing-risk analyses, the present findings should not be interpreted as supporting rosiglitazone use for TB prevention or as altering its current clinical risk–benefit assessment.

The differing associations observed for rosiglitazone and pioglitazone also raise questions regarding possible drug-specific mechanisms. Although both agents have broadly similar glucose-lowering efficacy, pioglitazone appears to exert greater effects on lipid profiles [28]. Experimental evidence further suggests that pioglitazone has partial PPARα activity, whereas rosiglitazone is a more selective and potent PPARγ agonist [15]. Given the role of PPARγ in regulating macrophage activation, cytokine production, and inflammatory signaling, differences in receptor selectivity may result in distinct immunomodulatory effects that could influence host defense against *M. tuberculosis* [13,29]. However, the mechanisms underlying the observed associations remain uncertain. Improved glycemic control may reduce TB susceptibility by mitigating hyperglycemia-associated immune dysfunction, whereas a direct PPARγ-mediated immunomodulatory effect is also biologically plausible. Although the differing associations between rosiglitazone and pioglitazone may suggest a potential drug-specific effect, this interpretation remains speculative. Because the NHIRD lacks HbA1c, blood glucose measurements, and immune biomarkers, differences in glycemic control could not be adequately accounted for, nor could glycemic benefits be distinguished from direct immunomodulatory effects. Therefore, the present study cannot determine whether the observed association was mediated by improved glycemic control, direct PPARγ-mediated immunomodulation, or both. In addition, rosiglitazone prescribing may have been influenced by evolving cardiovascular safety concerns, reimbursement policies, regulatory restrictions, and physician preferences. Despite extensive propensity score matching and multivariable adjustment, unmeasured differences in cardiovascular risk, frailty, metabolic characteristics, healthcare utilization, and treatment adherence may have contributed to the observed associations.

Experimental studies have suggested that PPARγ activation may influence host immune responses to *M. tuberculosis* through modulation of macrophage function, inflammatory cytokines, and innate immune signaling pathways [13,29,30,31,32,33]. These findings provide biological plausibility for a potential association between rosiglitazone use and reduced TB risk. However, the present observational study was not designed to investigate underlying biological mechanisms; therefore, these experimental findings should be regarded as hypothesis-generating rather than explanatory evidence.

Several limitations should be considered. First, TB was identified using ICD-9/10 diagnostic codes. Although previous validation studies have demonstrated good accuracy for TB diagnoses in the NHIRD [23], some misclassification remains possible. To improve specificity, anti-TB medication use and TB-related hospitalization were additionally considered. Nevertheless, the diagnosis-code-based pulmonary TB endpoint may have included provisional or suspected cases, and the absence of microbiological, radiographic, and detailed clinical data precluded assessment of diagnostic uncertainty or potential overdiagnosis. Information on latent TB infection and preventive therapy was also unavailable. The incidence of the primary outcome was lower than national TB estimates, possibly because of the stringent outcome definition and study selection criteria, including the exclusion of patients with heart failure, hepatic failure, or prior TB. Together with propensity score matching, these criteria may have resulted in a relatively healthier cohort. Accordingly, our findings should be interpreted primarily as relative risk comparisons rather than estimates of absolute TB incidence. Second, the primary analysis used an ITT-like, baseline-fixed exposure definition and therefore did not account for subsequent treatment discontinuation, switching between individual TZDs, or initiation of TZD therapy among baseline nonusers. These post-index treatment changes may have resulted in exposure misclassification. Although complementary time-dependent analyses were performed, participants were not censored specifically at treatment discontinuation or switching; therefore, these analyses did not constitute a strict as-treated analysis. Notably, the associations were attenuated in the time-dependent analyses, and none remained statistically significant after death was accounted for as a competing event. These findings suggest that differential survival and survivor/selection bias may have influenced the primary estimates. Future studies incorporating explicit treatment-continuation definitions, grace periods, censoring rules, and appropriate methods for time-varying treatment are warranted. Third, the NHIRD lacks reliable direct information on alcohol consumption, smoking behavior, dietary habits, nutritional status, physical activity, vaccination history, and important clinical and laboratory variables, including body mass index, hemoglobin A1c, blood glucose levels, microbiological findings, and immune markers. The low prevalence of obesity and smoking-related diagnoses likely reflects undercoding in administrative claims data and should not be interpreted as the true prevalence of these characteristics. Despite adjustment for DCSI scores, medication burden, and multiple comorbidities, residual confounding by unmeasured lifestyle, metabolic, and clinical factors remains possible. In particular, the absence of direct measures of glycemic control prevented us from determining whether the observed associations were related to improved glycemic control, direct immunomodulatory effects of rosiglitazone, or both. Furthermore, the NHIRD captures only diagnoses, procedures, and treatments documented by healthcare professionals, potentially introducing ascertainment and misclassification bias. Although nationwide coverage and validated diagnostic algorithms, including the high-specificity definition requiring both a TB diagnosis and anti-TB treatment, may mitigate this concern, such bias cannot be eliminated. Fourth, cumulative rosiglitazone exposure was analyzed as a time-fixed variable. Greater cumulative exposure may partly reflect longer survival and treatment persistence, thereby introducing survivor and healthy-adherer biases. In addition, several cumulative-duration categories contained relatively few events, resulting in limited statistical precision. Accordingly, the duration-related findings should be regarded as exploratory rather than evidence of a causal exposure–response relationship. Fifth, the study population consisted almost entirely of Taiwanese adults aged 20–80 years, which may limit the generalizability of our findings to other ethnic populations and individuals older than 80 years. Nevertheless, the findings may be relevant to regions with a high TB burden, particularly in Southeast Asia. Given the greater frailty, immunosenescence, and comorbidity burden among older adults, future studies should specifically evaluate the association between rosiglitazone use and TB risk in individuals aged >80 years. Sixth, multiple outcomes, drug-specific comparisons, cumulative duration analyses, subgroup analyses, and sensitivity analyses were performed without formal adjustment for multiple testing, increasing the possibility of type I error. These secondary and exploratory findings should therefore be interpreted cautiously and require independent confirmation. Finally, calendar-time confounding cannot be completely excluded. Rosiglitazone prescribing changed substantially during the study period in response to evolving cardiovascular safety concerns, regulatory restrictions, reimbursement policies, and physician preferences. Although index year was incorporated into the propensity score model and diabetes duration at cohort entry was aligned between matched groups, exact matching or stratification by calendar period was not performed. Thus, adjustment for index year may not have fully captured these temporal changes. Moreover, the longer mean follow-up among rosiglitazone users than nonusers (9.64 vs. 8.40 years) may partly reflect differences in treatment era and available follow-up time. Taken together with the attenuation of the associations in time-dependent analyses and their loss of statistical significance after accounting for competing mortality, these limitations raise the possibility that residual confounding, differential survival, survivor/selection bias, and temporal changes in treatment selection contributed to the primary findings. Accordingly, the observed associations should be regarded as hypothesis-generating rather than evidence of a causal or protective effect of rosiglitazone.

## 5. Conclusions

In this nationwide observational cohort study, rosiglitazone use was associated with lower risks of TB-related outcomes in the primary Cox analyses. However, these associations were attenuated in time-dependent analyses and were no longer statistically significant after death was accounted for as a competing event. The findings may therefore have been influenced by differential mortality, survivor/selection bias, treatment-selection effects, calendar-time confounding, and residual confounding from unmeasured clinical and lifestyle factors. Accordingly, these results should be regarded as hypothesis-generating associations rather than evidence of a causal or protective effect of rosiglitazone on TB risk. Further studies using more robust causal-inference approaches are needed to determine whether an independent association exists.

## Figures and Tables

**Table 1:** Baseline characteristics of matched patients according to rosiglitazone use.

Variables	Non-Rosiglitazone		Rosiglitazone		SMD
	(N = 9225)		(N = 9225)		
	n	%	n	%	
Sex					0.016
Female	4451	48.25	4525	49.05	
Male	4774	51.75	4700	50.95	
Age, years					
20–39	412	4.47	413	4.48	0.001
40–49	1098	11.90	1171	12.69	0.024
50–59	2185	23.69	2319	25.14	0.034
60–69	2841	30.80	2839	30.78	<0.0001
70–80	2689	29.15	2483	26.92	0.050
Mean (SD)	61.41	11.55	60.85	11.55	0.049
Comorbidities					
Obesity	128	1.39	122	1.32	0.006
Smoking	42	0.46	36	0.39	0.010
Alcohol-related disorders	247	2.68	238	2.58	0.006
Hypertension	6440	69.81	6444	69.85	0.001
Dyslipidemia	4808	52.12	4963	53.80	0.034
CAD	2884	31.26	2883	31.25	<0.0001
Stroke	1508	16.35	1489	16.14	0.006
PAD	740	8.02	764	8.28	0.010
CKD	476	5.16	481	5.21	0.002
COPD	1549	16.79	1573	17.05	0.007
Pneumonia	723	7.84	714	7.74	0.004
Liver cirrhosis	342	3.71	331	3.59	0.006
Rheumatologic diseases	148	1.60	139	1.51	0.008
Cancer	371	4.02	386	4.18	0.008
Psychosis	636	6.89	640	6.94	0.002
Depression	153	1.66	154	1.67	0.001
Dementia	52	0.56	39	0.42	0.020
Organ transplantation	10	0.11	13	0.14	0.009
CCI					
0	5239	56.79	5165	55.99	0.016
1	1317	14.28	1441	15.62	0.038
≥2	2669	28.93	2619	28.39	0.012
DCSI					
0	2288	24.80	2164	23.46	0.031
1	1733	18.79	1764	19.12	0.009
≥2	5204	56.41	5297	57.42	0.020
Medications					
Metformin	7622	82.62	7773	84.26	0.044
DPP-4 inhibitors	3890	42.17	4082	44.25	0.042
Sulfonylureas	9053	98.14	8979	97.33	0.054
AGI	4861	52.69	5135	55.66	0.060
SGLT2 inhibitors	430	4.66	476	5.16	0.023
Number of oral antidiabetic drugs					
0–1	146	1.58	201	2.18	0.044
2–3	3644	39.50	3285	35.61	0.080
>3	5435	58.92	5739	62.21	0.067
Insulin	8298	89.95	8306	90.04	0.003
Statin	5151	55.84	5123	55.53	0.006
Aspirin	2980	32.30	3145	34.09	0.038
Corticosteroid	4549	49.31	4590	49.76	0.009
Immunosuppressants	23	0.25	23	0.25	0.000

Note: SMD: standardization mean difference; SD, standard deviation; PAD, peripheral arterial disease; CKD, chronic kidney disease; COPD, chronic obstructive pulmonary disease; CCI, Charlson Comorbidity Index; DCSI, Diabetes Complications Severity Index; DPP-4, dipeptidyl peptidase-4; AGI, alpha-glucosidase inhibitors; SGLT2, sodium glucose cotransporter-2. SMDs are reported as absolute effect-size measures, with values < 0.10 generally indicating adequate balance between groups after propensity score matching.

**Table 2:** Risk of tuberculosis-related outcomes associated with use versus nonuse of rosiglitazone in patients with type 2 diabetes.

	Non-Rosiglitazone			Rosiglitazone								
Outcomes	N	PY	IR	N	PY	IR	cHR	(95% CI)	*p*-Value	aHR	(95% CI)	*p*-Value
TB plus receipt of anti-TB treatment	53	77,848	0.68	35	89,414	0.39	0.54	(0.35, 0.83)	0.0051	0.56	(0.36, 0.86)	0.0087
Pulmonary TB	381	76,766	4.96	329	87,991	3.74	0.77	(0.67, 0.9)	<0.001	0.79	(0.68, 0.92)	0.0018
TB-related hospitalization	93	77,841	1.19	65	89,376	0.73	0.6	(0.44, 0.83)	0.0018	0.59	(0.43, 0.81)	0.0012

Notes: TB, tuberculosis; PY, person–years; IR, incidence rate per 1000 person–years; cHR, crude hazard ratio; aHR: Adjusted hazard ratio derived from multivariable models controlling for sex, age, comorbidities, Charlson Comorbidity Index (CCI), Diabetes Complication Severity Index (DCSI), insulin use, statin use, aspirin use, and the type and number of oral antidiabetic agents, as detailed in Table 1.

**Table 3:** Risk of tuberculosis-related outcomes associated with the cumulative duration of rosiglitazone use.

Variables	Event	PY	IR	cHR	(95% CI)	*p* Value	aHR	(95% CI)	*p*-Value
TB plus receipt of anti-TB treatment									
Nonuse of rosiglitazone	53	77,848	0.68	1.00	(reference)	-	1.00	(reference)	-
Cumulative duration of rosiglitazone use (days)									
1–84	10	27,548	0.36	0.52	(0.27, 1.03)	0.0605	0.57	(0.29, 1.14)	0.1109
85–420	13	27,491	0.47	0.68	(0.37, 1.25)	0.2182	0.71	(0.38, 1.31)	0.2712
>420	12	34,376	0.35	0.46	(0.24, 0.85)	0.0143	0.45	(0.24, 0.85)	0.0139
Pulmonary TB									
Nonuse of rosiglitazone	381	76,766	4.96	1.00	(reference)	-	1.00	(reference)	-
Cumulative duration of rosiglitazone use (days)									
1–84	122	27,091	4.50	0.92	(0.75, 1.12)	0.3999	0.89	(0.73, 1.09)	0.2724
85–420	109	27,017	4.03	0.82	(0.66, 1.02)	0.0684	0.83	(0.67, 1.03)	0.0873
>420	98	33,883	2.89	0.62	(0.49, 0.77)	<0.001	0.66	(0.53, 0.82)	<0.001
TB-related hospitalization									
Nonuse of rosiglitazone	93	77,841	1.19	1.00	(reference)	-	1.00	(reference)	-
Cumulative duration of rosiglitazone use (days)									
1–84	21	27,546	0.76	0.64	(0.4, 1.03)	0.0641	0.59	(0.36, 0.95)	0.0298
85–420	16	27,478	0.58	0.49	(0.29, 0.83)	0.0076	0.48	(0.28, 0.82)	0.0068
>420	28	34,351	0.82	0.67	(0.44, 1.02)	0.0628	0.68	(0.44, 1.04)	0.0745

Notes: TB, tuberculosis; PY, person–years; IR, incidence rate per 1000 person–years; cHR, crude hazard ratio; aHR: Adjusted hazard ratio derived from multivariable models controlling for sex, age, comorbidities, Charlson Comorbidity Index (CCI), Diabetes Complication Severity Index (DCSI), insulin use, statin use, aspirin use, and the type and number of oral antidiabetic agents, as detailed in Table 1.

**Table 4:** Competing-risk analysis of tuberculosis-related outcomes associated with rosiglitazone use, with death treated as a competing event.

Outcome		sHR	95% CI	*p*-Value
TB plus receipt of anti-TB treatment	Non-rosiglitazone	1	(reference)	-
	Rosiglitazone	1.00	(0.76, 1.32)	0.983
Pulmonary TB	Non-rosiglitazone	1	(reference)	-
	Rosiglitazone	0.90	(0.71, 1.15)	0.399
TB-related hospitalization	Non-rosiglitazone	1	(reference)	-
	Rosiglitazone	0.86	(0.65, 1.13)	0.268

Notes: TB, tuberculosis; sHR, subdistribution hazard ratio; CI, confidence interval. Death was treated as a competing event using the Fine–Gray subdistribution hazard model. Adjusted sHRs were estimated after controlling for sex, age, comorbidities, Charlson Comorbidity Index (CCI), Diabetes Complication Severity Index (DCSI), insulin use, statin use, aspirin use, and the type and number of oral antidiabetic agents, as detailed in Table 1.

## Data Availability

The data used in this study were obtained from the National Health Insurance Research Database (NHIRD), maintained by Taiwan’s National Health Insurance (NHI) Administration. Due to Taiwan’s Personal Information Protection Act (Enforced since 2012), these data cannot be publicly shared in the paper, Supplementary Materials, or a public repository. Researchers interested in accessing the dataset can submit a formal request to the NHIRD Office (https://dep.mohw.gov.tw/DOS/cp-5119-59201-113.html, accessed on 1 July 2026) or by contacting stsung@mohw.gov.tw.

## Supplementary Materials

The following supporting information can be downloaded at: https://www.mdpi.com/article/10.3390/microorganisms14091979/s1. Table S1. Diagnostic conditions and corresponding ICD-9-CM and ICD-10-CM codes applied in this study. Table S2. Baseline characteristics of matched patients according to TZD use. Table S3. Risk of tuberculosis-related outcomes associated with use versus nonuse of TZDs, pioglitazone, and rosiglitazone in patients with type 2 diabetes. Table S4. Risk of TB-related outcomes associated with rosiglitazone use versus nonuse following exclusion of patients with tuberculosis-related events within 6 months after the index date. Table S5. Risk of TB-related outcomes associated with rosiglitazone use versus nonuse: results of time-dependent analysis. Table S6. Subgroup analysis of the risk of TB plus receipt of anti-TB treatment associated with rosiglitazone use versus nonuse among patients with type 2 diabetes. Table S7. Subgroup analysis of the risk of pulmonary TB associated with rosiglitazone use versus nonuse among patients with type 2 diabetes. Table S8. Subgroup analysis of the risk of TB-related hospitalization associated with rosiglitazone use versus nonuse among patients with type 2 diabetes. Figure S1. Love plots illustrating covariate balance before and after propensity score matching. Standardized mean differences (SMDs) for all 44 baseline covariates are shown before and after matching for the rosiglitazone cohort. After matching, all absolute SMDs were <0.1, indicating adequate covariate balance between the comparison groups. Figure S2. Restricted cubic spline analyses of the associations between cumulative rosiglitazone exposure and TB-related outcomes. Restricted cubic spline plots illustrate the continuous associations between cumulative duration of rosiglitazone exposure and the risks of (A) TB plus receipt of anti-TB treatment, (B) pulmonary TB, and (C) TB-related hospitalization. Figure S3. Forest plot summarizing subgroup analyses of the association between rosiglitazone use versus nonuse and the risk of TB plus receipt of anti-TB treatment. Squares indicate adjusted hazard ratios (aHRs), and horizontal lines represent 95% confidence intervals (CIs). Figure S4. Forest plot summarizing subgroup analyses of the association between rosiglitazone use versus nonuse and the risk of pulmonary TB. Squares indicate adjusted hazard ratios (aHRs), and horizontal lines represent 95% confidence intervals (CIs). Figure S5. Forest plot summarizing subgroup analyses of the association between rosiglitazone use versus nonuse and the risk of TB-related hospitalization. Squares indicate adjusted hazard ratios (aHRs), and horizontal lines represent 95% confidence intervals (CIs).

## Abbreviations

The following abbreviations are used in this manuscript:

TB	Tuberculosis
TZDs	Thiazolidinediones
T2D	Type 2 diabetes
RR	Relative risk
PPARγ	Peroxisome proliferator-activated receptor-γ
NHIRD	National Health Insurance Research Database
CCI	Charlson Comorbidity Index
DCSI	Diabetes Complication Severity Index
