# Supplementary material for: Association Between Thiazolidinedione Therapy and Tuberculosis-Related Events in Type 2 Diabetes: A Population-Based Cohort Study
Source: Microorganisms. 2026 Sep 8;14(9):1979. doi: 10.3390/microorganisms14091979 (PMC13609190; doi:10.3390/microorganisms14091979)
Supplement: Supplementary file 1 [file microorganisms-14-01979-s001.zip › microorganisms-4524855-supplementary.pdf]

Table S1. Diagnostic conditions and corresponding ICD-9-CM and ICD-10-CM codes applied in this study.

Disease	ICD-9-CM codes	ICD-10-CM codes
Alcohol-related disorders	291, 303, 305.0, 571.0-571.3, V11.3, V79.1	F10, K70.40, K70.41, K70.9
Coronary artery disease	410	I21, I22
Chronic obstructive pulmonary disease	491, 492, or 496	J41, J42, J44, J43, or J44.9
Cancers	140-208	C00-C96
Chronic kidney disease	250.4x, 403.xx, 404.xx, 585.xx, 586.xx, 581.8x,<br>791.0x, 593.9x, V56.0, V56.8, V45.1	E10.2, E10.65, E11.2, E11.65, E13.2, I12, I13, N03, N08, E10.21, E11.21, N05, N06, N07, N14, N15.0, N15.8, N15.9, N16, N17.1, N17.2, N18, N19, Z49.31, Z49.32, Z99.2
Dyslipidemia	272	E71.30, E71.31, E71.32, E71.39, E75.21, E75.22, E75.23, E75.24, E75.25, E75.29, E75.3, E75.4, E75.5, E75.6, E77, E78.0, E78.1, E78.2, E78.3, E78.4, E78.5, E78.6, E78.70, E78.71, E78.72, E78.79, E78.8, E78.9
Depression	311	F32, F33
Dementia	290,290.4, 291.2, 292.82, 331	F03.90, F05, F02.80, F02.81, F01.50, F01.51, G30
Hypertension	401–405, A26	I10, I11, I12, I13, I15, N26
Heart failure	428	I50
Hepatic failure	570, 572.2, 572.4, 572.8	K72.00, K72.01, K72.10, K72.11, K72.90, K76.2, K72.90, K72.91, K76.7, K76.81
Liver cirrhosis	571.5, 571.2, 571.6	K70.2, K70.30, K70.31, K74.0, K74.1, K74.2, K74.60, K74.69, K74.3, K74.4, K74.5
Obesity	278.02, 783.1, V85.2, 278.00, 649.1, V77.8, V85.3, 278.01, 649.2, V45.86, V85.4	R63.5, E66.09, E66.1, E66.8, E66.9, Z13.89, E66.01, E66.2
Organ transplantation	V42	Z94
Pneumonia	480-486	J12-18

Peripheral artery disease	440.0, 440.20, 440.21, 440.22, 440.23, 440.24, 440.3, 440.4, 443.9, 443.81, 443.89	I70.2, I70.92, I75.0, I73.9
Psychosis	290–299	F20-29
Pulmonary tuberculosis	011	A15.0
Rheumatologic diseases	710.0, 710.1, 710.4, 714.0–714.2, 714.81, 725	M05-08, M12, M32-36
Smoking	305.1, 649.0, V15.82	F17.200, F17.201, F17.210, F17.220, F17.221, F17.290, F17.291, Z87.891
Stroke	430-438	G45.0, G45.1, G45.2, G45.3, G45.4, G45.8, G45.9, G46, I60, I61, I62, I63, I65, I66, I67.0, I67.1, I67.2, I67.3, I67.4, I67.5, I67.6, I67.7, I67.8, I67.9, I68, I69
Type 2 diabetes mellitus	250.xx, except 250.1x	E11
Type 1 diabetes mellitus	250.1x	E10
Tuberculosis	010- 018	A15, A17, A18, A19

Table S2. Baseline characteristics of matched patients according to TZD use.

**Variables**	**Non-TZD**		**TZD**		**SMD**
	**(N=44095)**		**(N=44095)**		
	**n**	**%**	**n**	**%**	
Sex					0.006
Female	19966	45.28	20107	45.60	
Male	24129	54.72	23988	54.40	
Age, years					
20-39	2755	6.25	2347	5.32	0.040
40-49	6551	14.86	6336	14.37	0.014
50-59	12803	29.04	13320	30.21	0.026
60-69	12771	28.96	13397	30.38	0.031
70-80	9215	20.90	8695	19.72	0.029
Mean (SD)	61.16	9.28	60.7	8.63	0.012
Comorbidities					
Obesity	1463	3.32	1444	3.27	0.002
Smoking	1163	2.64	1188	2.69	0.004
Alcohol-related disorders	1540	3.49	1635	3.71	0.012
Hypertension	30866	70.00	30841	69.94	0.001
Dyslipidemia	30757	69.75	30485	69.13	0.013
CAD	12293	27.88	12399	28.12	0.005
Stroke	5569	12.63	5739	13.02	0.012
PAD	3681	8.35	3762	8.53	0.007
CKD	2462	5.58	2506	5.68	0.004
COPD	7207	16.34	7257	16.46	0.003
Pneumonia	4264	9.67	4257	9.65	0.001
Liver cirrhosis	1233	2.80	1282	2.91	0.007
Rheumatologic diseases	804	1.82	806	1.83	<0.001
Cancer	1961	4.45	2011	4.56	0.005
Psychosis	3005	6.81	3121	7.08	0.010
Depression	1190	2.70	1179	2.67	0.002
Dementia	96	0.22	113	0.26	0.008
Organ transplantation	62	0.14	47	0.11	0.010
CCI index					
0	25849	58.62	25367	57.53	0.022
1	7745	17.56	8245	18.70	0.029
≥2	10501	23.81	10483	23.77	0.001
DCSI					
0	11788	26.73	11571	26.24	0.011
1	8572	19.44	8691	19.71	0.007
≥2	23735	53.83	23833	54.05	0.004
Medications					
Metformin	39039	88.53	39055	88.57	0.001
DPP-4 inhibitors	26886	60.97	26680	60.51	0.010

Sulfonylureas	41285	93.63	41465	94.04	0.017
AGI	19699	44.67	19378	43.95	0.015
SGLT2 inhibitors	5433	12.32	5299	12.02	0.009
Number of oral antidiabetic drugs					
0-1	1248	2.83	1117	2.53	0.018
2-3	15942	36.15	15909	36.08	0.002
>3	26905	61.02	27069	61.39	0.008
Insulin	32254	73.15	32618	73.97	0.019
Aspirin	23073	52.33	23284	52.80	0.010
Statin	23196	52.60	22876	51.88	0.015
Corticosteroid	29876	67.75	29806	67.59	0.003
Immunosuppressants	128	0.29	119	0.27	0.004

Note: TZD, thiazolidinedione; SMD, standardization mean difference; SD, standard deviation; PAD, peripheral arterial disease; CKD, chronic kidney disease; COPD, chronic obstructive pulmonary disease; CCI, Charlson Comorbidity Index; DCSI, Diabetes Complications Severity Index; DPP-4, dipeptidyl peptidase-4; AGI, alpha-glucosidase inhibitors; SGLT2, sodium glucose cotransporter-2.

Table S3. Risk of tuberculosis-related outcomes associated with use versus nonuse of TZDs, pioglitazone, and rosiglitazone in patients with type 2 diabetes.

	**TB plus receipt of anti-TB treatment**								
**Variables**	**Event**	**PY**	**IR**	**cHR**	**(95% CI)**	**p-value**	**aHR**	**(95% CI)**	**p-value**
TZD									
No	178	262405	0.68	1.00	(reference)	-	1.00	(reference)	-
Yes	235	294416	0.80	1.15	(0.94, 1.39)	0.1709	1.16	(0.95, 1.41)	0.1422
Pioglitazone									
No	203	320783	0.63	1.00	(reference)	-	1.00	(reference)	-
Yes	210	236038	0.89	1.44	(1.19, 1.75)	<0.001	1.43	(1.18, 1.74)	<0.001
Rosiglitazone									
No	337	421212	0.80	1.00	(reference)	-	1.00	(reference)	-
Yes	76	135610	0.56	0.56	(0.43, 0.72)	<0.001	0.57	(0.44, 0.74)	<0.001
	**Pulmonary TB**								
**Variables**	**Event**	**PY**	**IR**	**cHR**	**(95% CI)**	**p-value**	**aHR**	**(95% CI)**	**p-value**
TZD									
No	733	260562	2.81	1.00	(reference)	-	1.00	(reference)	-
Yes	814	291797	2.79	1	(0.91, 1.11)	0.9343	0.98	(0.89, 1.08)	0.6738
Pioglitazone									
No	978	317944	3.08	1.00	(reference)	-	1.00	(reference)	-
Yes	569	234416	2.43	0.79	(0.71, 0.87)	<0.001	0.92	(0.83, 1.03)	0.1418
Rosiglitazone									

No	1080	418681	2.58	1.00	(reference)	-	1.00	(reference)	-
Yes	467	133678	3.49	1.47	(1.31, 1.64)	<0.001	1.09	(0.97, 1.22)	0.1469

	**TB-related hospitalization**								
**Variables**	**Event**	**PY**	**IR**	**cHR**	**(95% CI)**	**p-value**	**aHR**	**(95% CI)**	**p-value**
TZD									
No	212	262573	0.81	1.00	(reference)	-	1.00	(reference)	-
Yes	211	294816	0.72	0.86	(0.71, 1.04)	0.1289	0.85	(0.7, 1.03)	0.0929
Pioglitazone									
No	262	320901	0.82	1.00	(reference)	-	1.00	(reference)	-
Yes	161	236488	0.68	0.86	(0.7, 1.04)	0.1203	0.93	(0.77, 1.14)	0.5064
Rosiglitazone									
No	326	421752	0.77	1.00	(reference)	-	1.00	(reference)	-
Yes	97	135637	0.72	0.74	(0.59, 0.94)	0.013	0.66	(0.52, 0.84)	<0.001

Notes: TZD, thiazolidinedione; TB, tuberculosis; PY, person-years; IR, incidence rate, per 1000 person-years; cHR, crude hazard ratio; aHR, adjusted hazard ratio.

aHR: Adjusted hazard ratio derived from multivariable models controlling for sex, age, comorbidities, Charlson Comorbidity Index (CCI), Diabetes Complication Severity Index (DCSI), insulin use, statin use, aspirin use, and the type and number of oral antidiabetic agents, as detailed in Supplementary Table S2.

Table S4. Risk of TB-related outcomes associated with rosiglitazone use versus nonuse following exclusion of patients with tuberculosis-related events within 6 months after the index date.

	**Non-rosiglitazone**			**Rosiglitazone**			**cHR**	**95%CI**	**P value**	**aHR**	**95%CI**	**p-value**
	**N**	**PY**	**IR**	**N**	**PY**	**IR**						
TB plus receipt of anti-TB treatment	103	31116	3.31	99	43678	2.27	0.69	(0.52, 0.91)	0.009	0.66	(0.49, 0.89)	0.007
Pulmonary TB	139	31023	4.48	119	43574	2.73	0.62	(0.48, 0.79)	<0.001	0.6	(0.46, 0.78)	<0.001
TB-related hospitalization	106	31174	3.4	95	43750	2.17	0.64	(0.48, 0.84)	0.001	0.59	(0.44, 0.80)	<0.001

Notes: TB, tuberculosis; PY, person-years; IR, incidence rate, per 1000 person-years; cHR, crude hazard ratio; aHR, adjusted hazard ratio.
aHR: Adjusted hazard ratio derived from multivariable models controlling for sex, age, comorbidities, Charlson Comorbidity Index (CCI), Diabetes Complication Severity Index (DCSI), insulin use, statin use, aspirin use, and the type and number of oral antidiabetic agents, as detailed in Table 1.

Table S5. Risk of TB-related outcomes associated with rosiglitazone use versus nonuse: results of time-dependent analysis.

**Variable**		**cHR**	**95%CI**	**P value**	**aHR**	**95%CI**	**p-value**
TB plus receipt of anti-TB treatment	Non-rosiglitazone	1	(reference)	-	1	(reference)	-
	Rosiglitazone	0.79	(0.40, 1.56)	0.495	0.82	(0.42, 1.62)	0.569
Pulmonary TB	Non-rosiglitazone	1	(reference)	-	1	(reference)	-
	Rosiglitazone	0.78	(0.44, 1.38)	0.389	0.82	(0.46, 1.46)	0.500
TB-related hospitalization	Non-rosiglitazone	1	(reference)	-	1	(reference)	-
	Rosiglitazone	0.48	(0.23, 0.98)	0.044	0.49	(0.24, 0.99)	0.050

Notes: TB, tuberculosis; PY, person-years; IR, incidence rate, per 1000 person-years; cHR, crude hazard ratio; aHR, adjusted hazard ratio.
aHR: Adjusted hazard ratio derived from multivariable models controlling for sex, age, comorbidities, Charlson Comorbidity Index (CCI), Diabetes Complication Severity Index (DCSI), insulin use, statin use, aspirin use, and the type and number of oral antidiabetic agents, as detailed in Table 1.

Table S6. Subgroup analysis of the risk of TB plus receipt of anti-TB treatment associated with rosiglitazone use versus nonuse among patients with type 2 diabetes.

Variable	Non-rosiglitazone			Rosiglitazone			cHR	95%CI	P value	aHR	95%CI	p-value
	N	PY	IR	N	PY	IR						
Age < 65	52	19465	2.67	46	27783	1.66	0.65	(0.43, 0.96)	0.032	0.59	(0.38, 0.91)	0.018
Age≧65	60	11740	5.11	62	15931	3.89	0.77	(0.54, 1.09)	0.142	0.71	(0.48, 1.04)	0.076
Female	38	16065	2.37	25	22041	1.13	0.49	(0.30, 0.82)	0.006	0.44	(0.26, 0.76)	0.003
Male	74	15140	4.89	83	21673	3.83	0.8	(0.59, 1.10)	0.174	0.77	(0.55, 1.08)	0.135
COPD												
No	84	25406	3.31	82	35311	2.32	0.71	(0.52, 0.97)	0.029	0.67	(0.48, 0.93)	0.018
Yes	28	5799	4.83	26	8403	3.09	0.66	(0.39, 1.13)	0.131	0.56	(0.32, 1.00)	0.048
CCI												
0-1	60	21378	2.81	59	25914	2.28	0.81	(0.57, 1.17)	0.262	0.81	(0.54, 1.20)	0.289
≧2	52	9826	5.29	49	17801	2.75	0.55	(0.37, 0.82)	0.003	0.53	(0.35, 0.79)	0.002
DCSI												
0-1	47	14372	3.27	47	21078	2.23	0.69	(0.46, 1.03)	0.069	0.64	(0.41, 1.01)	0.058

≧2	65	16833	3.86	61	22636	2.69	0.72	(0.51, 1.03)	0.071	0.65	(0.45, 0.94)	0.023
OAD												
0-1	86	24660	3.49	80	33371	2.4	0.7	(0.51, 0.95)	0.021	0.67	(0.48, 0.94)	0.019
≧2	26	6545	3.97	28	10343	2.71	0.71	(0.41, 1.21)	0.207	0.58	(0.33, 1.02)	0.058
Insulin												
No	78	21952	3.55	74	31159	2.37	0.68	(0.49, 0.93)	0.018	0.64	(0.45, 0.91)	0.012
Yes	34	9253	3.67	34	12555	2.71	0.77	(0.48, 1.24)	0.284	0.68	(0.41, 1.14)	0.141
Corticosteroids												
No	39	9550	4.08	32	13374	2.39	0.59	(0.37, 0.95)	0.029	0.52	(0.31, 0.88)	0.014
Yes	73	21654	3.37	76	30340	2.5	0.77	(0.55, 1.06)	0.107	0.75	(0.53, 1.06)	0.101

Notes: TB, tuberculosis; PY, person-years; IR, incidence rate, per 1000 person-years; cHR, crude hazard ratio; aHR, adjusted hazard ratio. COPD, chronic obstructive pulmonary disease; OAD, oral antidiabetic drugs.

aHR: Adjusted hazard ratio derived from multivariable models controlling for sex, age, comorbidities, Charlson Comorbidity Index (CCI), Diabetes Complication Severity Index (DCSI), insulin use, statin use, aspirin use, and the type and number of oral antidiabetic agents, as detailed in Table 1.

Table S7. Subgroup analysis of the risk of pulmonary TB associated with rosiglitazone use versus nonuse among patients with type 2 diabetes.

Variable	Non-rosiglitazone			Rosiglitazone			cHR	95%CI	P value	aHR	95%CI	p-value
	N	PY	IR	N	PY	IR						
Age < 65	57	19440	2.93	62	27695	2.24	0.8	(0.56, 1.15)	0.228	0.72	(0.48, 1.06)	0.093
Age≧65	97	11656	8.32	71	15889	4.47	0.55	(0.40, 0.74)	<0.001	0.53	(0.38, 0.73)	<0.001
Female	55	16005	3.44	30	22014	1.36	0.41	(0.26, 0.64)	<0.001	0.38	(0.24, 0.62)	<0.001
Male	99	15091	6.56	103	21570	4.78	0.75	(0.57, 0.99)	0.046	0.72	(0.54, 0.97)	0.032
COPD												
No	109	25356	4.3	100	35233	2.84	0.67	(0.51, 0.88)	0.005	0.66	(0.49, 0.89)	0.006
Yes	45	5740	7.84	33	8350	3.95	0.53	(0.34, 0.83)	0.006	0.46	(0.29, 0.75)	0.002
CCI												
0-1	81	21334	3.8	69	25866	2.67	0.71	(0.52, 0.98)	0.038	0.73	(0.51, 1.05)	0.087
≧2	73	9763	7.48	64	17717	3.61	0.51	(0.37, 0.72)	<0.001	0.5	(0.35, 0.71)	<0.001
DCSI												
0-1	61	14323	4.26	55	21026	2.62	0.62	(0.43, 0.90)	0.011	0.64	(0.42, 0.96)	0.03
≧2	93	16774	5.54	78	22558	3.46	0.65	(0.48, 0.88)	0.005	0.58	(0.42, 0.80)	<0.001
OAD												

0-1	122	24564	4.97	96	33298	2.88	0.6	(0.46, 0.78)	<0.001	0.6	(0.45, 0.80)	<0.001
≧2	32	6533	4.9	37	10286	3.6	0.76	(0.47, 1.21)	0.248	0.6	(0.36, 0.99)	0.047
Insulin												
No	105	21880	4.8	87	31095	2.8	0.6	(0.45, 0.80)	<0.001	0.58	(0.43, 0.79)	<0.001
Yes	49	9216	5.32	46	12489	3.68	0.72	(0.48, 1.07)	0.108	0.64	(0.42, 0.98)	0.041
Corticosteroids												
No	56	9500	5.89	36	13343	2.7	0.46	(0.31, 0.71)	<0.001	0.42	(0.27, 0.68)	<0.001
Yes	98	21597	4.54	97	30241	3.21	0.74	(0.55, 0.98)	0.033	0.72	(0.54, 0.98)	0.035

Notes: TB, tuberculosis; PY, person-years; IR, incidence rate, per 1000 person-years; cHR, crude hazard ratio; aHR, adjusted hazard ratio. COPD, chronic obstructive pulmonary disease; OAD, oral antidiabetic drugs.

aHR: Adjusted hazard ratio derived from multivariable models controlling for sex, age, comorbidities, Charlson Comorbidity Index (CCI), Diabetes Complication Severity Index (DCSI), insulin use, statin use, aspirin use, and the type and number of oral antidiabetic agents, as detailed in Table 1.

Table S8. Subgroup analysis of the risk of TB-related hospitalization associated with rosiglitazone use versus nonuse among patients with type 2 diabetes.

Variable	Non-rosiglitazone			Rosiglitazone			cHR	95%CI	P value	aHR	95%CI	p-value
	N	PY	IR	N	PY	IR						
Age < 65	47	19508	2.41	48	27822	1.73	0.74	(0.49, 1.10)	0.14	0.62	(0.40, 0.96)	0.033
Age≧65	72	11751	6.13	54	15995	3.38	0.55	(0.39, 0.78)	<0.001	0.52	(0.36, 0.76)	<0.001
Female	38	16095	2.36	28	22045	1.27	0.55	(0.33, 0.89)	0.016	0.52	(0.31, 0.89)	0.016
Male	81	15164	5.34	74	21772	3.4	0.64	(0.47, 0.89)	0.007	0.58	(0.41, 0.81)	0.001
COPD												
No	82	25508	3.21	75	35395	2.12	0.66	(0.48, 0.91)	0.01	0.6	(0.42, 0.84)	0.003
Yes	37	5751	6.43	27	8422	3.21	0.51	(0.31, 0.83)	0.008	0.45	(0.27, 0.76)	0.003
CCI												
0-1	59	21415	2.76	57	25984	2.19	0.8	(0.55, 1.15)	0.219	0.78	(0.52, 1.16)	0.222
≧2	60	9844	6.1	45	17833	2.52	0.43	(0.29, 0.63)	<0.001	0.4	(0.27, 0.59)	<0.001
DCSI												
0-1	46	14415	3.19	46	21115	2.18	0.69	(0.45, 1.03)	0.071	0.63	(0.40, 1.00)	0.052
≧2	73	16844	4.33	56	22702	2.47	0.58	(0.41, 0.83)	0.003	0.52	(0.36, 0.74)	<0.001
OAD												
0-1	94	24683	3.81	71	33488	2.12	0.56	(0.41, 0.76)	<0.001	0.53	(0.38, 0.74)	<0.001
≧2	25	6576	3.8	31	10329	3	0.82	(0.48, 1.39)	0.466	0.6	(0.34, 1.04)	0.07
Insulin												
No	83	21971	3.78	66	31263	2.11	0.56	(0.41, 0.78)	<0.001	0.52	(0.37, 0.74)	<0.001

Yes	36	9287	3.88	36	12554	2.87	0.76	(0.48, 1.20)	0.24	0.59	(0.36, 0.97)	0.037
Corticosteroids												
No	42	9583	4.38	27	13432	2.01	0.46	(0.28, 0.74)	0.002	0.34	(0.20, 0.59)	<0.001
Yes	77	21675	3.55	75	30385	2.47	0.71	(0.52, 0.98)	0.037	0.68	(0.49, 0.96)	0.028

Notes: TB, tuberculosis; PY, person-years; IR, incidence rate, per 1000 person-years; cHR, crude hazard ratio; aHR, adjusted hazard ratio. COPD, chronic obstructive pulmonary disease; OAD, oral antidiabetic drugs.

aHR: Adjusted hazard ratio derived from multivariable models controlling for sex, age, comorbidities, Charlson Comorbidity Index (CCI), Diabetes Complication Severity Index (DCSI), insulin use, statin use, aspirin use, and the type and number of oral antidiabetic agents, as detailed in Table 1.

Supplementary Figure Legends

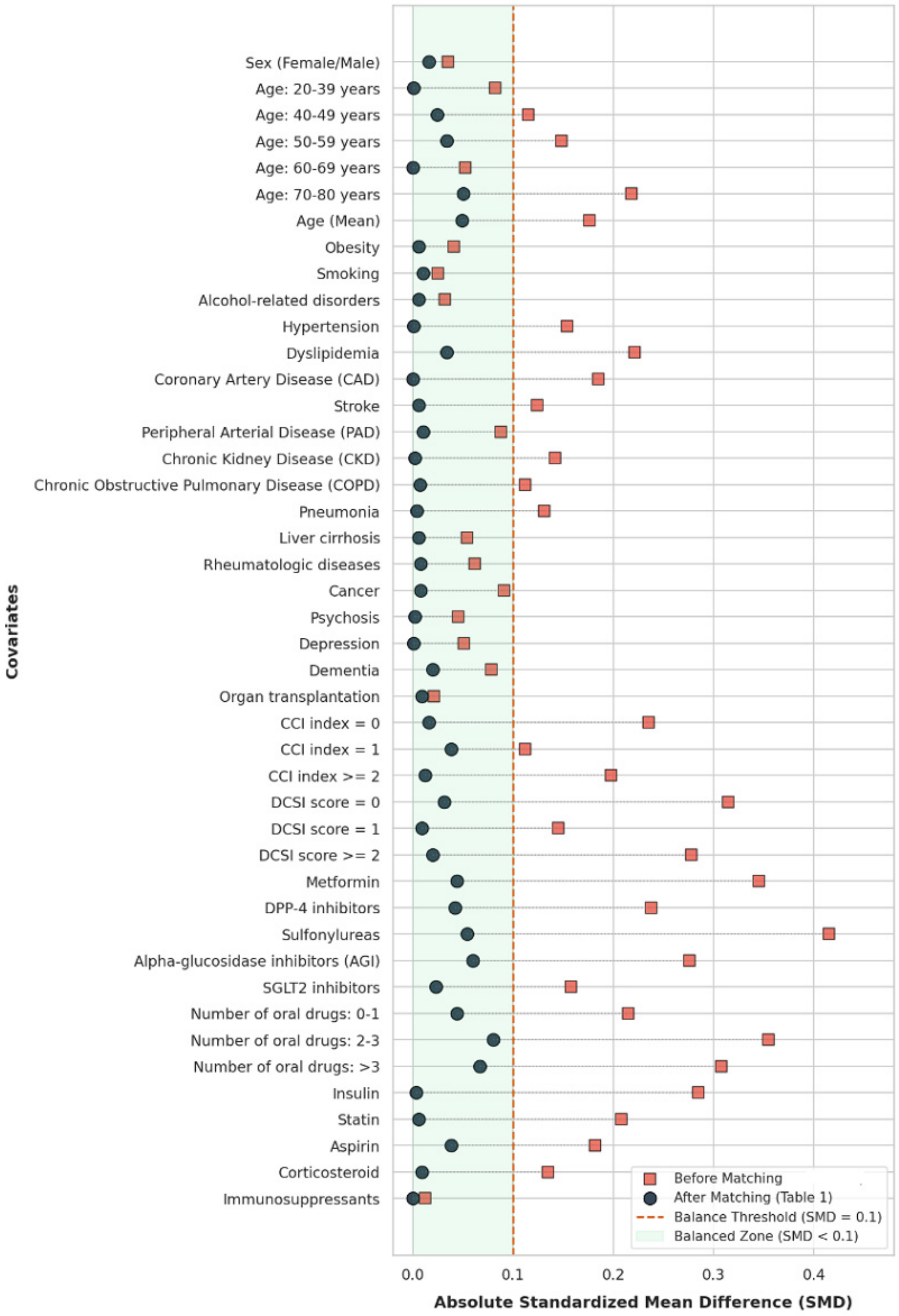

Figure S1. Love plots illustrating covariate balance before and after propensity score matching. Standardized mean differences (SMDs) for all 44 baseline covariates are

shown before and after matching for the rosiglitazone cohort. After matching, all absolute SMDs were <0.1, indicating adequate covariate balance between the comparison groups.

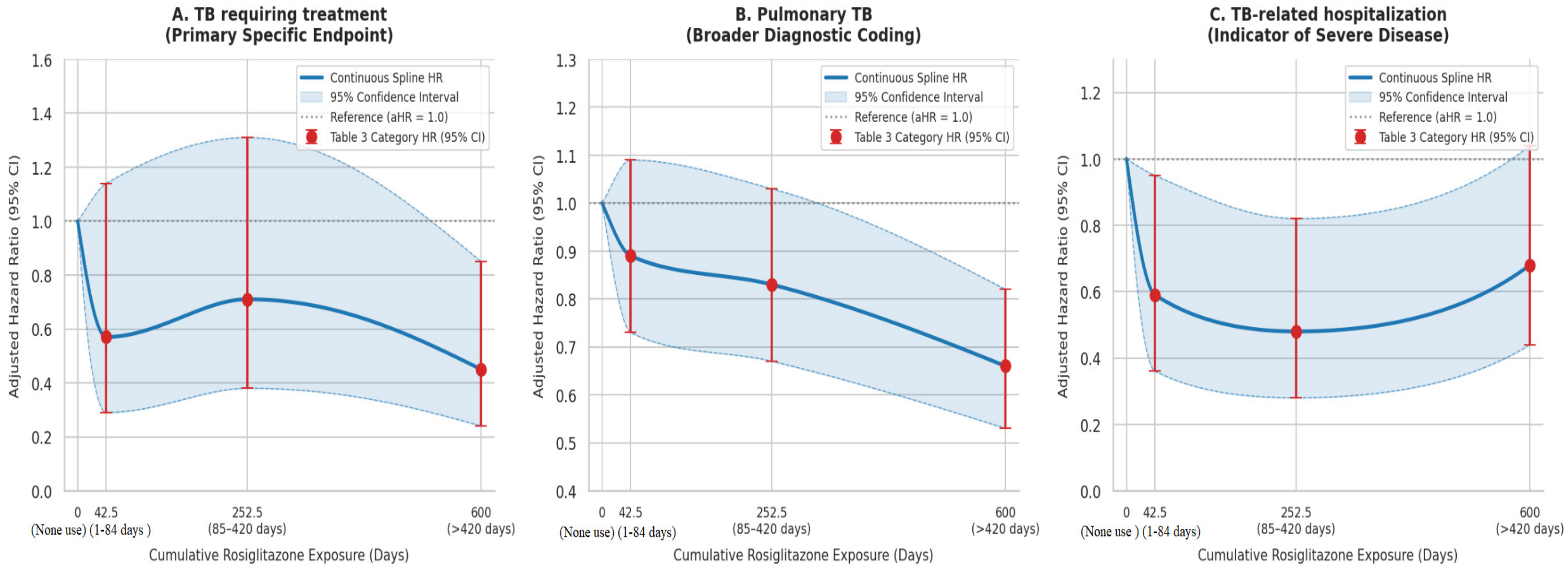

Figure S2. Restricted cubic spline analyses of the associations between cumulative rosiglitazone exposure and TB-related outcomes. Restricted cubic spline plots illustrate the continuous associations between cumulative duration of rosiglitazone exposure and the risks of (A) TB plus receipt of anti-TB treatment, (B) pulmonary TB, and (C) TB-related hospitalization.

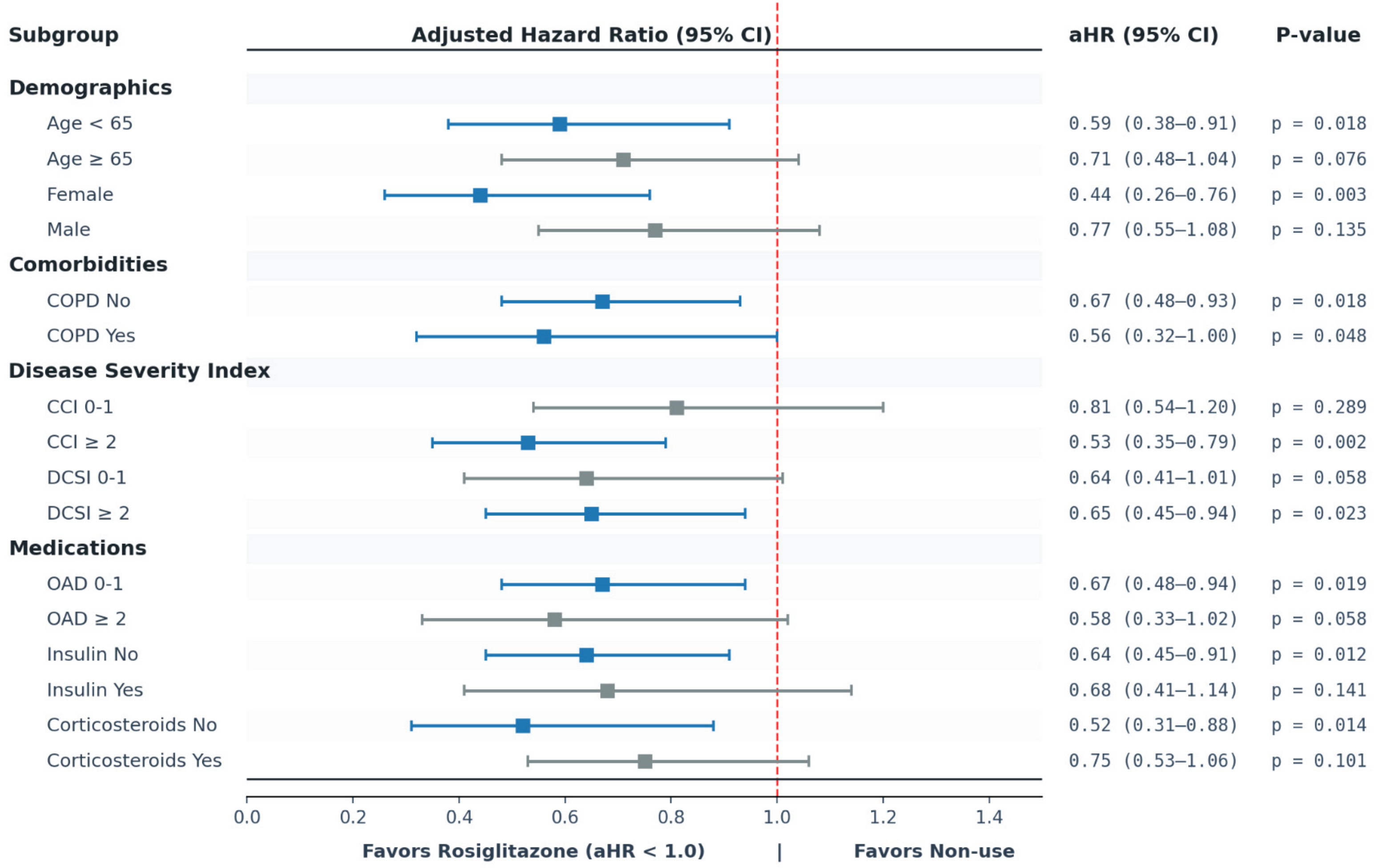

Figure S3. Forest plot summarizing subgroup analyses of the association between rosiglitazone use versus nonuse and the risk of TB plus receipt of anti-TB treatment. Squares indicate adjusted hazard ratios (aHRs), and horizontal lines represent 95% confidence intervals (CIs).

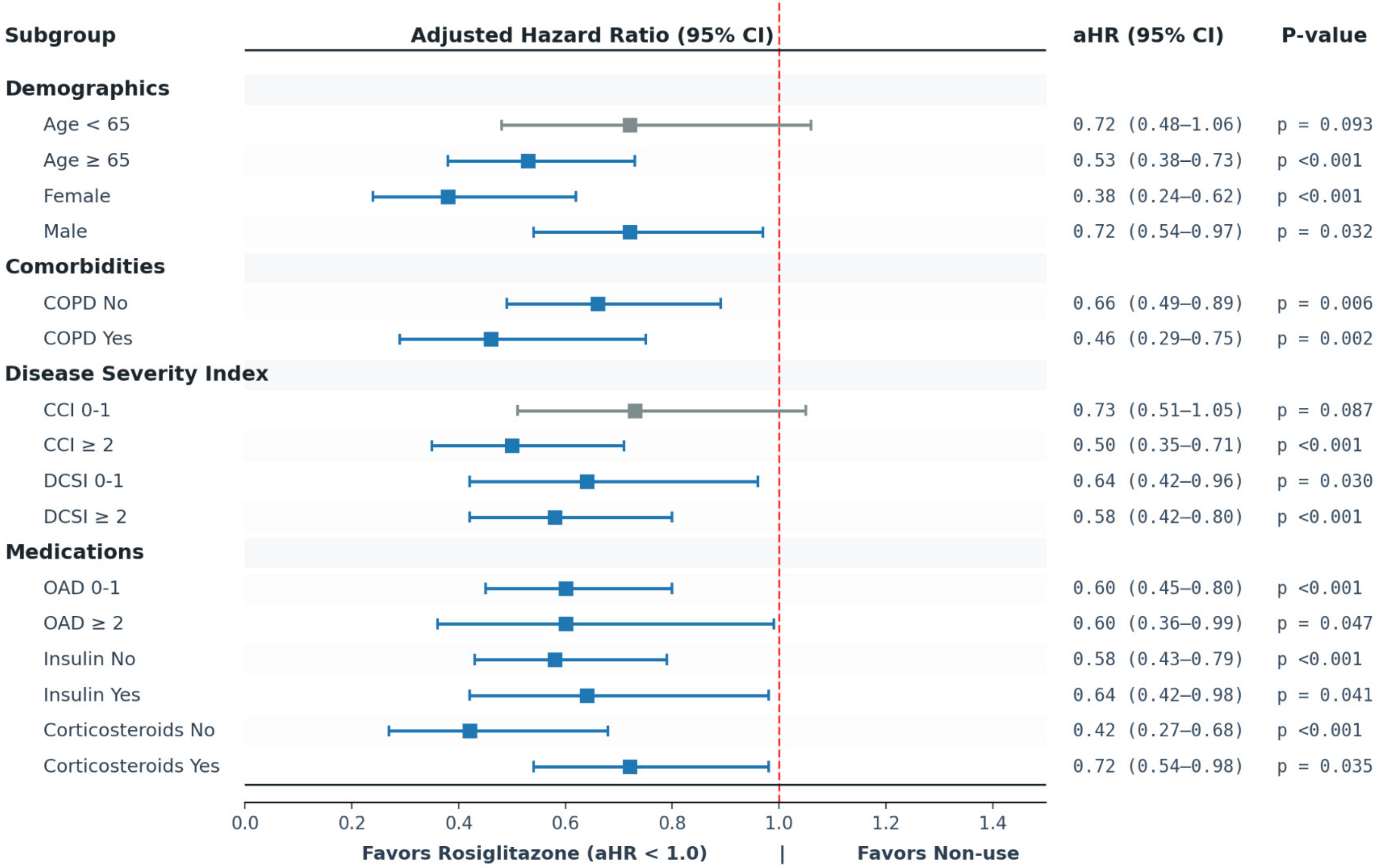

Figure S4. Forest plot summarizing subgroup analyses of the association between rosiglitazone use versus nonuse and the risk of pulmonary TB. Squares indicate adjusted hazard ratios (aHRs), and horizontal lines represent 95% confidence intervals (CIs).

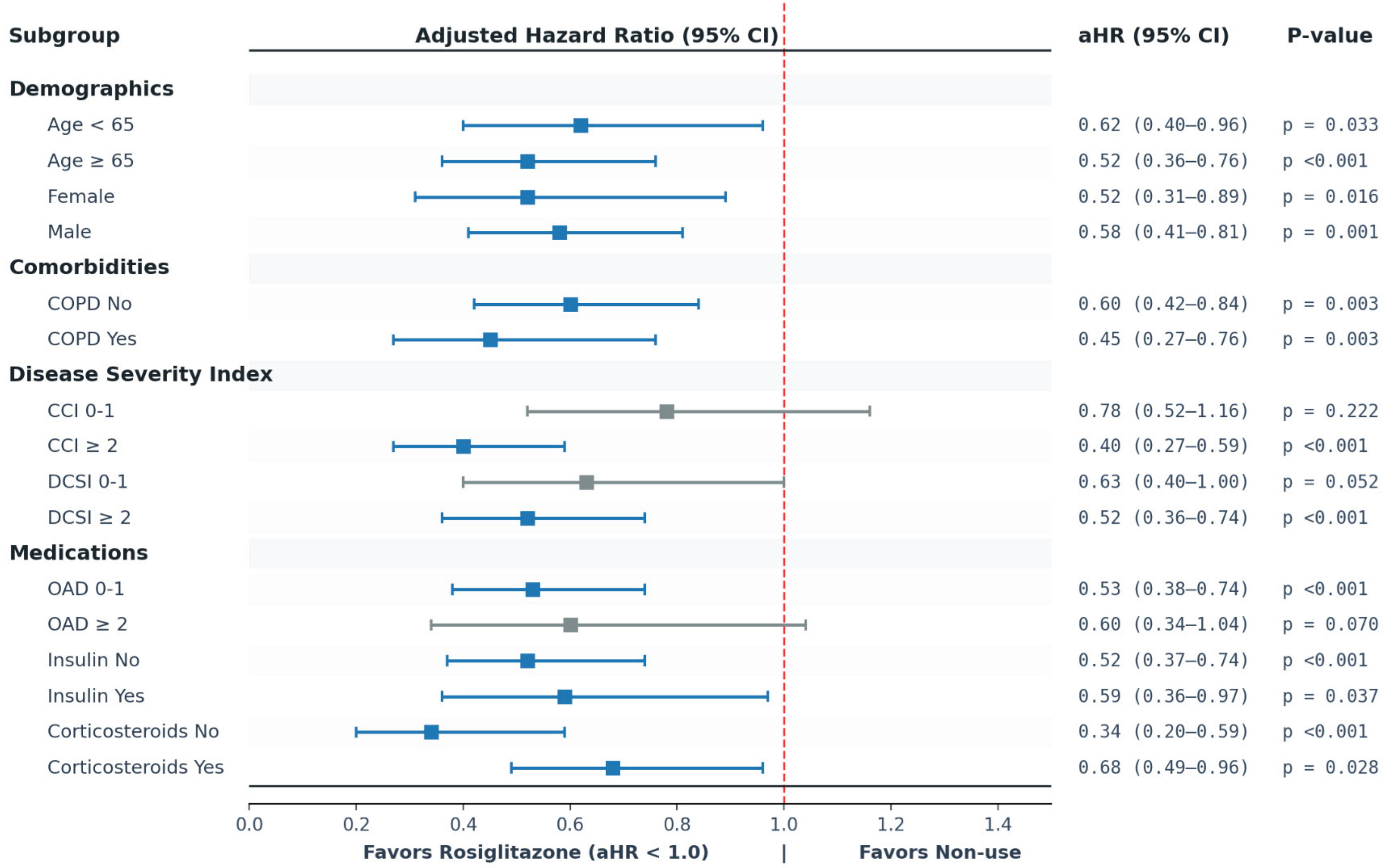

Figure S5. Forest plot summarizing subgroup analyses of the association between rosiglitazone use versus nonuse and the risk of TB-related hospitalization. Squares indicate adjusted hazard ratios (aHRs), and horizontal lines represent 95% confidence intervals (CIs).
